# Development and Validation of a Liquid Chromatography-Tandem Mass Spectrometry Method Coupled with Dispersive Solid-Phase Extraction for Simultaneous Quantification of Eight Paralytic Shellfish Poisoning Toxins in Shellfish

**DOI:** 10.3390/toxins9070206

**Published:** 2017-06-29

**Authors:** Xianli Yang, Lei Zhou, Yanglan Tan, Xizhi Shi, Zhiyong Zhao, Dongxia Nie, Changyan Zhou, Hong Liu

**Affiliations:** 1 Institute for Agro-food Standards and Testing Technology, Laboratory of Quality & Safety Risk Assessment for Agro-products (Shanghai), Ministry of Agriculture, Shanghai Academy of Agricultural Sciences, Shanghai 201403, China; zhaozhiyong7097@163.com (Z.Z.); niedongxia@163.com (D.N.); changyanz@sina.com (C.Z.); 2 School of Marine Sciences, Ningbo University, Ningbo 315211, China; zhoulei736371@163.com (L.Z.); shixizhi@nbu.edu.cn (X.S.); 3 Key Laboratory of Food Safety Research, Institute for Nutritional Sciences, Shanghai Institutes for Biological Sciences, Chinese Academy of Sciences, Shanghai 200031, China; yanglantan@gmail.com; 4 Shanghai Municipal Center for Disease Control and Prevention, Shanghai 200336, China

**Keywords:** paralytic shellfish poisoning (PSP) toxins, LC-MS/MS, dispersive solid-phase extraction (dSPE), saxitoxin

## Abstract

In this study, a high-performance liquid chromatography-tandem mass spectrometry (HPLC-MS/MS) method was developed for simultaneous determination of eight paralytic shellfish poisoning (PSP) toxins, including saxitoxin (STX), neosaxitoxin (NEO), gonyautoxins (GTX1–4) and the *N*-sulfo carbamoyl toxins C1 and C2, in sea shellfish. The samples were extracted by acetonitrile/water (80:20, *v*/*v*) with 0.1% formic and purified by dispersive solid-phase extraction (dSPE) with C18 silica and acidic alumina. Qualitative and quantitative detection for the target toxins were conducted under the multiple reaction monitoring (MRM) mode by using the positive electrospray ionization (ESI) mode after chromatographic separation on a TSK-gel Amide-80 HILIC column with water and acetonitrile. Matrix-matched calibration was used to compensate for matrix effects. The established method was further validated by determining the linearity (*R*^2^ ≥ 0.9900), average recovery (81.52–116.50%), sensitivity (limits of detection (LODs): 0.33–5.52 μg·kg^−1^; limits of quantitation (LOQs): 1.32–11.29 μg·kg^−1^) and precision (relative standard deviation (RSD) ≤ 19.10%). The application of this proposed approach to thirty shellfish samples proved its desirable performance and sufficient capability for simultaneous determination of multiclass PSP toxins in sea foods.

## 1. Introduction

The paralytic shellfish poisoning (PSP) toxins are potent neurotoxins produced by toxic algae in red tide [1,2] and further accumulated in shellfish [3,4]. They are known for causing severe food poisoning by intake of contaminated shellfish or other seafood [5]. These naturally produced toxins show thermal stability and cannot be destroyed even under normal heating. At least 57 saxitoxin (STX) analogues congeners were identified and typically classified into four groups [6]. STX is considered as the most important toxin in the known PSP toxins [1,2]. According to Oshima, the toxicity of the other PSP toxins is calculated in relation to STX [7]. The regulatory limit for PSP toxins in shellfish meat has been set at 800 μg STXequ·kg^−1^ [8]. A joint report by the Food and Agriculture Organization (FAO), the World Health Organization (WHO) and the International Oceanographic Commission of United Nations Educational, Scientific and Cultural Organization (IOC) has recommended a derived guidance level of 170 or 110 μg STXequ·kg^−1^ based on the consumption of 250 or 380 g shellfish meat by adults [9], and then a level of 75 μg STXequ·kg^−1^ based on 400 g feed size was suggested [10].

**Figure 1:**
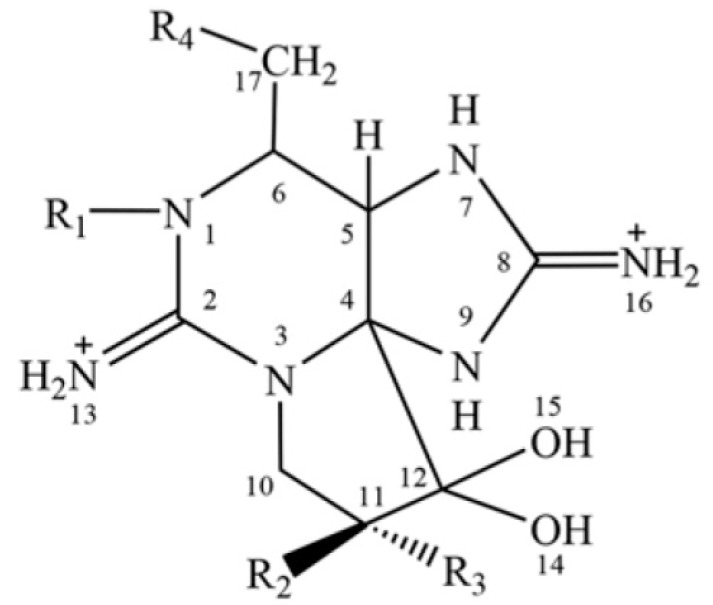
Chemical structures of eight target paralytic shellfish poisoning (PSP) toxins.

Eight commonly occurring toxins (Figure 1), including STX, neosaxitoxin (neoSTX, NEO), gonyautoxins (GTX1–4) and the N-sulfocarbamoyl toxins C1 and C2, were studied in this work. The carbamate toxins, including STX, NEO and GTX1–4, are more potent than C1 and C2. However, C1 and C2 can be changed into the other highly toxic molecules form induced by the environment or biological transformation [6], indicating that the monitoring of lowly toxic C1 and C2 should not be neglected. The AOAC (Association of Official Analytical Chemists) mouse bioassay (MBA) was applied for routine monitoring of PSP toxins [11] with a specified reference method by European Union (EU) legislation [12]. However, this method only provides a total toxicity value without information about specific toxin profiles of a real sample. Also, MBA has the disadvantages of poor sensitivity, low stability and interferences from other compounds in the matrix.

Another concern about analysis is that generally PSP toxins do not have natural ultraviolet or fluorescence absorption and thus need derivation pretreatments before detection by liquid chromatography. Post-column derivatization [13,14,15,16] or pre-column derivatization [17,18,19,20] with fluorescence detection (LC-FLD) shows high sensitivity but requires a complex equipment with daily maintenance. In recent years, the development methods of liquid chromatography-tandem mass spectrometry (LC-MS/MS) for determination of cyanobacterial toxins and STXs were carried out in previous studies [21,22,23,24,25] with a hydrophilic interaction liquid chromatography (HILIC)-based column for separation. This type of column was proved to give good retention of the PSP toxins [26,27,28,29] and thus applied in our study.

Matrix interferences are common problems that occur when using LC-MS/MS. The appropriate sample pretreatment technique, for example solid-phase extraction (SPE), has reduced the interferences effectively. Also, the dispersive solid-phase extraction (dSPE) has been applied as a cleanup procedure prior to LC-MS/MS [30,31,32]. Compared to the conventional SPE, dSPE has the advantages of fast and simple operation [33], minimal solvent consumption and low costs [30], as well as reducing matrix interferences and increasing column lifetime [34]. It is important to choose appropriate adsorbent materials to purify the sample matrixes and thus minimize the matrix interference in dSPE techniques. In the study, some dSPE adsorbents materials were evaluated and applied for cleanup procedures. Also, an LC-MS/MS method coupled with dSPE was developed for simultaneous determination of eight PSP toxins in shellfish.

## 2. Results and Discussion

### 2.1. Optimization of HPLC-MS/MS Conditions

The mobile phase included organic phase and aqueous phase. For reversed-phase LC, acetonitrile and methanol are commonly used as organic phase, while acetonitrile was considered for better resolution than methanol in some of the previous reports [22]. However, acetonitrile is always selected as organic phase in HILIC studies. In addition, pH of water phase could affect the retention time, peak shape and method sensitivity. Herein, formic acid and ammonium formate buffer were applied to adjust pH which was set to 2.7, 3.0, 3.3, 3.5 and 4.0, respectively. The result indicated that retention time and separation efficiency increased with increased pH. The chromatographic peaks of C1/C2/GTX2 were not separated completely under pH 2.7 and 3.0, which did not allow accurate quantification. Besides, the baseline noise increased significantly and the sensitivity was reduced at pH 4.0. Consequently, pH 3.5 was finally chosen in this study. Although shown in previous study [30], addition of buffer in the organic phase could maintain the pH of mobile phase and ensure the stability of retention time. However, our experimental results indicated that the retention time, peak shape and sensitivity were not improved by adding buffer in organic phase, so pure acetonitrile was adopted in this study.

**Figure 2:**
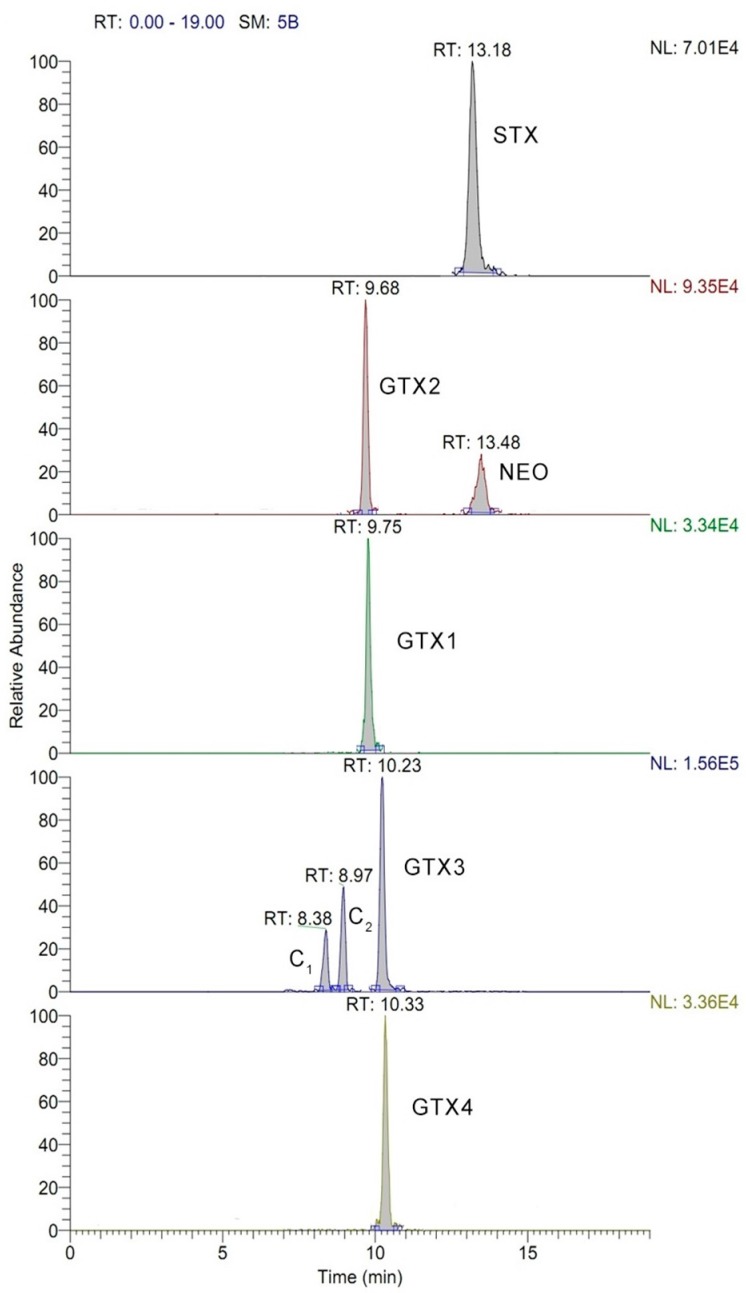
Extracted ion chromatograms of eight target PSP toxins in standard solution (STX: 19.98 ng·mL^−1^; NEO: 20.68 ng·mL^−1^; GTX1: 24.84 ng·mL^−1^; GTX2: 45.14 ng·mL^−1^; GTX3: 19.16 ng·mL^−1^; GTX4: 8.103 ng·mL^−1^; C1: 53.92 ng·mL^−1^; C2: 16.12 ng·mL^−1^). RT = retention time, SM = smooth point, NL = normalized level.

The MS/MS conditions were optimized for each PSP toxin with direct injection of the mixed stock solution. Precursor ions were observed by Q1 full scan in the positive mode, and the MS/MS conditions in the multiple reaction monitoring (MRM) mode were further optimized. The results showed that abundant [M + H]^+^ ions were generated from STX, NEO, GTX3 and GTX4. [M + H-SO_3_]^+^ions produced by GTX1, GTX2, C1 and C2 were obviously higher than their [M + H]^+^ ions, indicating in-source fragmentation [M + H]^+^ tend to lose neutral group SO_3_ and form stable [M + H-SO_3_]^+^ under the positive electrospray ionization (ESI) condition. Collision energies of precursor–product ion transitions were optimized to give the maximum intensity of the product ions. The precursor–product ion transition with the highest signal abundance was selected for quantification, while the secondary product ion and the abundance ration of two transitions were used for qualification. The optimized MS/MS parameters for the eight PSP toxins in MRM mode are shown in Table 1, and the extracted ion chromatograms of toxins is shown in Figure 2.

### 2.2. Optimization of Sample Pretreatment

**Figure 3:**
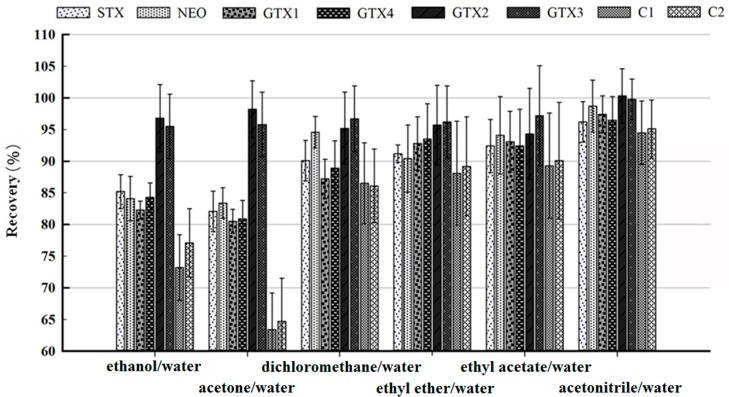
Extraction efficiencies of different extraction solvents for eight target PSP toxins.

Extraction solvent was optimized by evaluating recoveries of analytes which was affected by extraction capacities and possibly matrix effects with difference solvents [35]. In this study, in order to select suitable extraction solvents to achieve satisfactory recoveries, a variety of solvents acidified with 0.1% formic acid were evaluated by spiking the blank sample with the intermediate concentration (1/1000 of the mixed stock solution concentration), including (1) ethanol/water (80/20, *v/v*); (2) acetone/water (80/20, *v/v*); (3) dichloromethane/water (80/20, *v/v*); (4) ethyl ether/water (80/20, *v/v*); (5) ethyl acetate/water (80/20, *v/v*); and (6) acetonitrile/water (80/20, *v/v*). Each extraction solvent was tested in blank sample, and each experiment was done in triplicate, and a total of 18 samples were ran in the experiment. The extraction efficiencies of each extraction solvent are averaged and shown in Figure 3. Desirable recoveries were obtained ranging from 80.53 to 94.30% when selecting acetonitrile/water (80/20, *v/v*), so it was employed in the subsequent toxin extraction.

**Figure 4:**
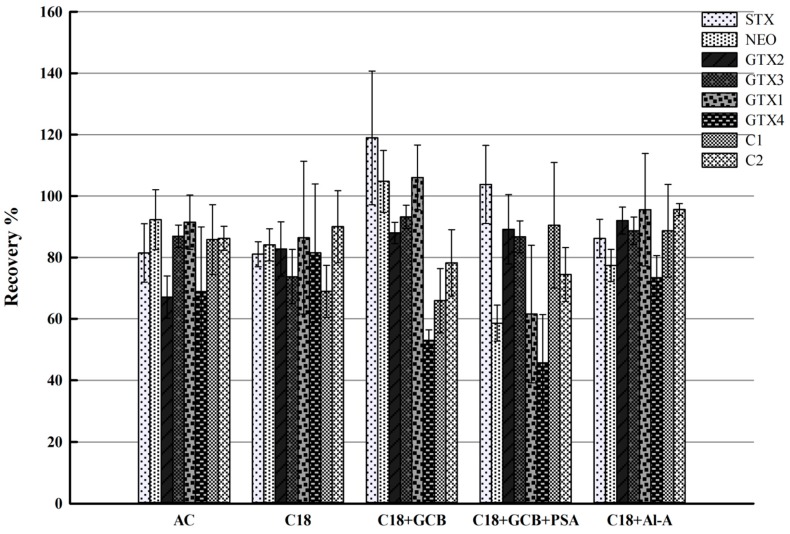
Recovery efficiencies of different dispersive solid-phase extraction (dSPE) adsorbents for eight target PSP toxins.

dSPE adsorbents were applied for purification to minimize the matrix effects. C18, a hydrophobic silica-based sorbent, was used commonly because of its strong affinity with non-polar compounds. Graphitized carbon black (GCB), which has a high affinity with planar molecules, is suitable for purification procedures, especially removal of the pigment and sterols. Ethylenediamine-N-propyl-silane (PSA) with two amino groups can remove pigment and metal ions. Activated carbon (AC) is capable of cleaning up impurities with negative charge by ion exchange. Acidic alumina (Al-A) was selected here as well, purification of PSP toxins by using merely Al-A has been reported [36]. According to the previous report [30], 100 mg of adsorbent was adequate for purification of 1g of sample. Various adsorbents or adsorbent mixtures, C18 (100 mg), AC (100 mg), C18 (90 mg) + GCB (10 mg), C18 (46.5 mg) + GCB (7 mg) + PSA (46.5 mg) and C18 (50 mg) + Al-A (50 mg) were thoroughly compared with regard to the recovery efficiencies in blank sample with the spiked intermediate concentration (1/1000 of the mixed stock solution concentration). Each experiment was performed in triplicate. The recoveries of PSP toxins by using above dSPE sorbents were shown in Figure 4. The recoveries, for example some high values obtained with C18 + GCB, also result from matrix effects after purification of adsorbents. Although highest recovery of each analyte was achieved with different sorbent material, acceptable recoveries (>70%) were presented for all of the eight PSP toxins only with C18 + Al-A, showing that the performance of C18 + Al-A was apparently a good choice and thus used as dSPE adsorbent in this study.

The composition of reconstitution solvent was considered to directly affect the peak shape and separation of the analytes in chromatographic system. In order to optimize the sample solvent, methanol, methanol-water (50/50, *v*/*v*), methanol-water (50/50, *v*/*v*) containing 10 mmol·L^−1^ ammonium acetate, methanol-water (50/50, *v*/*v*) containing 0.1% of formic acid and 0.1% formic acid aqueous solution were compared in the pilot test. The results showed that the best peak shapes were obtained with water with 0.1% formic acid, so it was applied as sample solvent in this study.

### 2.3. Evaluation of the Matrix Effects

**Figure 5:**
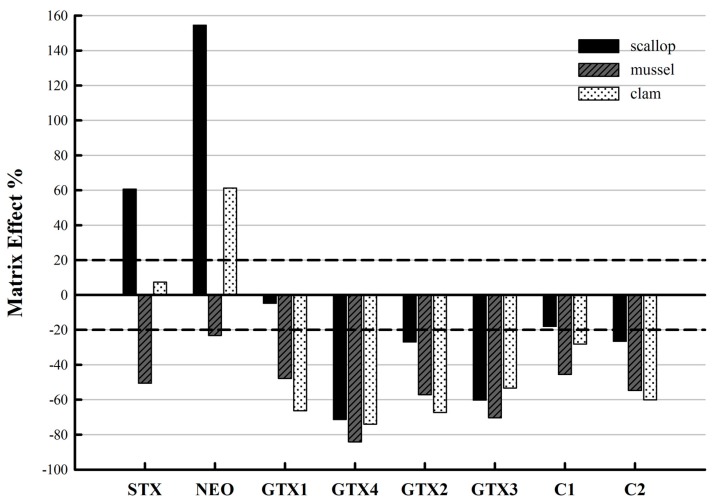
Matrix effects of eight target PSP toxins in the three matrices. A tolerance level of matrix effect was shown between the two dashed lines.

Matrix effects (ME) are a common problem that occurs when using LC-MS/MS. The extent of the signal suppression/enhancement (SSE) was observed differently for the eight PSP toxins. The SSE range between −20% and +20% is typically considered as tolerable [37]. The signals were generally suppressed, except signal enhancement of STX and NEO with the sample matrix scallop, mussel and clam. As shown in Figure 5, with a tolerance level indicated, significant matrix effects were observed. Since the matrix effects of the shellfish could interfere with method accuracy, the matrix-matched calibration curves by using analyte-free matrixes were further constructed for analysis to compensate the matrix effects and ensure the accurate quantification.

### 2.4. Method Validation

According to the EU guideline [38], method validation including the determination of recovery, precision, linearity, the limit of detection (LOD) and the limit of quantitation (LOQ), etc. was carried out.

As shown in Table 2, the matrix-matched calibration curves of toxins showed good linear relationships with coefficients of determination *R*^2^ ≥ 0.9900. The LODs and LOQs of this established method were in the range of 1.32–11.29 µg·kg^−1^ and 0.33–5.52 µg·kg^−1^. LOQs of STX (2.48 µg·kg^−1^), NEO (4.14 µg·kg^−1^), GTX1 (8.28 µg·kg^−1^), GTX2 (9.03µg·kg^−1^), GTX3 (4.79 µg·kg^−1^) and GTX4 (4.05 µg·kg^−1^) in clam were obviously lower than those of STX (11.3 µg·kg^−1^), NEO (19.0 µg·kg^−1^), GTX1 (26.7 µg·kg^−1^), GTX2 (38.3 µg·kg^−1^), GTX3 (17.3 µg·kg^−1^) and GTX4 (5.30 µg·kg^−1^) previously reported [30]. The results indicated that the proposed method has effectively reduced the matrix effect and has increased the sensitivity of the detection.

The results showed that the recoveries were acquired in the range of 81.52–116.50% for all tested PSP toxins in scallop, clam and mussel, as displayed in Table 3. Intra- and inter-day precision was investigated by spiking the analyte-free samples with three levels of the mixed standards and performing analysis on five consecutive days with six replicates every day. As shown in Table 3, the relative standard deviation (RSD) of intra-day was ranging from 1.50% to 19.10%, and RSD of inter-day was in the range of 0.35–18.37%. The results indicated that this established method was precise and can be adopted for analysis.

### 2.5. Sample Analysis

The validated method was employed to assess natural occurrence of STX, NEO, GTX1, GTX2, GTX3, GXT4, C1 and C2 in shellfish in China. The samples were collected and pretreated as described in Section 2.3 and Section 2.4. In the analysis, the matrix-matched calibration was applied to compensate the matrix effect and ensure the accurate quantification of the target toxins. As shown in Table 4, in a total of the collected thirty shellfish samples determined to contain PSP toxins (36.7% of incidence), ranging from 6.34 to 20.82 μg·kg^−1^, 6 of the 11 positive-result samples originate from western coastal waters of the Yellow Sea, which was consistent with previous reports [39].

## 3. Conclusions

The occurring of PSP toxins poses health risks and attracts attention in the system of seafood safety management. In the current study, HPLC-MS/MS method, coupled with simplified extraction and effective dSPE pretreatment procedures using C18 + Al-A as cleanup adsorbents, has been established for simultaneous quantification of STX, NEO, GTX1, GTX2, GTX3, GXT4, C1 and C2 in shellfish. After validation by investigating the sensitivity, linearity, accuracy, stability and matrix effects, the developed analytical method was applied to quantification of the eight toxins in shellfish samples collected from various areas of coastal waters in China. The results have proved that this method is reliable and practical for rapid detection of multiclass PSP toxins, and therefore suitable for both research and routine monitoring of PSP toxins in shellfish regarding the fields of fisheries and marine environment.

## 4. Materials and Methods

### 4.1. Materials and Reagents

The PSP toxin standards, including STX, NEO, GTX1–3, GTX2–4 and C1-2, were purchased from the National Research Council, Halifax, NS, Canada. Acetonitrile, formic acid and ammonium formate of HPLC grade were obtained from Merck (Darmstadt, Germany). Deionized water was purified using a Milli-Q Gradient A 10 System (Millipore, Billerica, MA, USA). All other chemicals and solvents were of HPLC or analytical grade. Acidic alumina was purchased from Sinopharm Chemical Reagent Co., Ltd. (Shanghai, China) and other dSPE adsorbents were purchased from Bonna-Agela Technologies Inc. (Wilmington, DE, USA).

### 4.2. Preparation of Standard Solutions

Accurately weighed solid portions of STX, NEO, GTX1, GTX2, GTX3, GXT4, C1 and C2 were dissolved with 0.1% formic acid aqueous solution to prepare stock solutions, and the concentrations of stock solutions were 198.44 μg·mL^−1^ (STX), 206.84 μg·mL^−1^ (NEO), 248.43 μg·mL^−1^ (GTX1), 451.43 μg·mL^−1^ (GTX2), 191.56 μg·mL^−1^ (GTX3), 81.03 μg·mL^−1^ (GTX4), 539.10 μg·mL^−1^ (C1) and 161.16 μg·mL^−1^ (C2), respectively. A mixed stock solution containing 39.69 μg·mL^−1^ of STX, 41.37 μg·mL^−1^ of NEO, 49.69 μg·mL^−1^ of GTX1, 90.29 μg·mL^−1^ of GTX2, 38.31 μg·mL^−1^ of GTX3, 16.21 μg·mL^−1^ of GXT4, 107.82 μg·mL^−1^ of C1 and 32.23 μg·mL^−1^ of C2 was prepared in 0.1% formic acid solution. All solutions were stored at −20 °C in the darkness. The working solutions were prepared from the mixed stock solution and stepwise diluted with 0.1% formic acid aqueous solution immediately before use. When the analyte-free shellfish were pretreated as described in Section 4.4, the working solutions were used to re-dissolve dried matrix residues for preparing matrix-matched standard solutions. Herein, analyte-free scallop (*Patinopecten yessoensis*), clam (*Meretrix lusoria*) and mussel (*Mytilus corusc*) were adopted for blank matrixes.

### 4.3. Samples

Thirty shellfish samples, including scallop, clam and mussel were purchased from local supermarkets in Shanghai city, and the origin information of the samples were collected. The soft tissues of the samples were homogenized by IKA T25 high-speed homogenizer (Ika-Werke GmbH, Staufen, Germany), and stored at −20 °C until further treatment.

### 4.4. Sample Pretreatment

Homogenate (1 ± 0.02 g) was weighted directly in a centrifuge tube and then extracted two times with 2 mL of acetonitrile/water (80:20, *v*/*v*) containing 0.1% formic acid. The combined extracting solution was vortex-mixed, ultrasonicated for 10 min and then centrifuged with 4500 rpm at 14 °C for 15 min. According to the previous study [30], after the supernatant was frozen under −20 °C for at least 4 h, the upper layer was removed quickly within 1 min. The remaining aqueous-phase layer was freeze-dried to near dryness, and then re-dissolved to 1 mL with water containing 0.1% formic acid. After mixing with 50 mg (±1 mg) C18 silica and 50 mg (±1 mg) acidic alumina (Al-A), the suspension was vortexed for 1 min and then centrifuged at 4500 rpm under less than 15 °C for 10 min. The supernatant was filtrated through a 0.22 μm nylon filter (ANPEL Technologies Inc., Shanghai, China) and ready for injection.

### 4.5. LC-MS/MS Analysis

The standard and pretreated sample solutions were directly injected into a Thermo Scientific triple quadrupole LC-MS/MS system (TSQ QUANTUM ULTRA, Thermo Scientific, San Jose, CA, USA) with positive-mode electrospray ionization (ESI) source and analysis was performed by using multiple reaction monitoring (MRM) mode. Separation was achieved on a TSK-gel Amide-80 HILIC column (Tosoh Bioscience Shanghai Co. Ltd., Shanghai, China) (150 mm × 2 mm, 3 μm) at 30 °C, with a mobile phase flow rate of 0.3 mL min^−1^. The mobile phase consisted of (A) formic acid (3.6 mM) and ammonium formate (2 mM) buffer solution and (B) acetonitrile. A linear gradient elution program was applied as follows: initial 15% A, 10 min 15% A, 11 min 45% A, 14 min 15% A, and finally 5 min for re-equilibration, giving a total run time of 19 min. The injection volume was 5.0 μL. The MS/MS settings were used as follows: spray voltage, 4.0 kV; vaporizer temperature, 300 °C; sheath gas pressure, 35 psi; aux valve flow, 20arb; capillary temperature, 350 °C. Data acquisition and processing were carried out using Thermo Xcalibur Series software (version1.3, Thermo Scientific, Waltham, MA, USA).

### 4.6. Evaluation of Matrix Effects

The evaluation of ME was based on by comparing the slope of the standard curves prepared in pure solvent and matrix-matched blank extract. The formula was as follows: ME (%) = [(slope of spiked extract/slope of pure standard) − 1] × 100%. ME of 0% indicated that no matrix effect occurred in the selected concentration ranges. ME above 0% revealed signal enhancement, while ME below 0% indicated signal suppression. The blank matrix was prepared using the analyte-free shellfish by following the sample pretreatment procedures. The mixed stock solutions were diluted with the blank matrix to yield a serial of concentrations (4/1000, 2/1000, 1/1000, 1/1500, 1/2000 and 1/4000 of stock solution concentration), respectively.

### 4.7. Validation of the Method

The parameters evaluated in the validation were linearity, precision (expressed as RSD in terms of intra-day and inter-day precision) and recovery. LODs were determined by measurements with successive dilution of matrix-spiked standard solution until a signal-to-noise ratio (S/N) of 3:1, and LOQs were defined as the concentration of a PSP toxin producing S/N = 10:1. Recovery experiments were performed in the analyte-free shellfish by employing the method of standard addition with low (9.92 for STX, 10.34 for NEO, 12.42 for GTX1, 22.57 for GTX2, 9.58 for GTX3, 4.05 for GTX4, 26.96 for C1 and 8.06 for C2 ng·mL^−1^), intermediate (26.46 for STX, 27.58 for NEO, 33.12 for GTX1, 60.19 for GTX2, 25.54 for GTX3, 10.80 for GTX4, 71.88 for C1 and 21.49 for C2 ng·mL^−1^) and high concentration (49.61 for STX, 51.71 for NEO, 62.11 for GTX1, 112.86 for GTX2, 47.89 for GTX3, 20.25 for GTX4, 134.78 for C1 and 40.29 for C2 ng·mL^−1^) of the mixed standards. Intra- and inter-day precision was investigated by spiking the analyte-free samples with three levels of the mixed standards and performing analysis on five consecutive days with six replicates every day.

## Figures and Tables

**Table:** 

Toxins	R_1_	R_2_	R_3_	R_4_
STX	H	H	H	OCONH_2_
NEO	OH	H	H	OCONH_2_
GTX1	OH	H	OSO_3_	OCONH_2_
GTX2	H	H	OSO_3_	OCONH_2_
GTX3	H	OSO_3_	H	OCONH_2_
GTX4	OH	OSO_3_	H	OCONH_2_
C1	H	H	OSO_3_	OCONHSO_3_
C2	H	OSO_3_	H	OCONHSO_3_

**Table 1:** MS/MS parameters for the eight target PSP toxins in multiple reaction monitoring (MRM) mode.

PSP Toxins	Transition Made from	Retention Time (min)	Precursor Ion (m/z)	Product Ion (m/z)	CE (eV)
C1	[M + H-SO_3_]^+^	8.38	396.1	316.1 *	13.0
				298.1	19.0
C2	[M + H-SO_3_]^+^	8.97	396.1	298.1 *	19.0
				316.1	13.0
GTX2	[M + H-SO_3_]^+^	9.68	316.1	220.0 *	26.0
				147.9	24.0
GTX1	[M + H-SO_3_]^+^	9.75	332.1	236.1 *	29.0
				164.0	32.0
GTX3	[M + H]^+^	10.23	396.1	298.1 *	19.0
				316.1	13.0
GTX4	[M + H]^+^	10.33	412.2	314.2 *	22.0
				332.2	21.0
STX	[M + H]^+^	13.18	300.1	204.0 *	25.0
				282.1	19.0
NEO	[M + H]^+^	13.48	316.1	220.1 *	21.0
				298.1	18.0

* Quantitative fragment ion.

**Table 2:** The matrix-matched calibration curves, linearity range and sensitivities of eight PSP toxins in three different matrixes (limit of detection (LOD) & limit of quantitation (LOQ): µg·kg^−1^).

PSP Toxins	Linearity Range (ng·mL^−1^)	Scallop			Mussel			Clam		
		*R*^2^	LOD	LOQ	*R*^2^	LOD	LOQ	*R*^2^	LOD	LOQ
STX	9.92~158.75	0.9982	0.33	1.32	0.9995	1.65	4.96	0.9986	0.82	2.48
NEO	10.34~165.47	0.9983	0.69	2.07	0.9982	2.59	5.17	0.9985	2.07	4.14
GTX1	12.42~198.74	0.9991	4.14	8.28	0.9960	5.52	8.28	0.9971	5.52	8.28
GTX2	22.57~361.15	0.9991	2.01	9.03	0.9959	2.82	11.29	0.9977	2.01	9.03
GTX3	9.58~153.25	0.9999	2.55	6.39	0.9937	4.79	9.58	0.9996	1.60	4.79
GTX4	4.05~64.82	0.9994	2.70	4.05	0.9957	3.04	6.08	0.9966	2.70	4.05
C1	26.96~431.28	0.9954	2.27	9.08	0.9977	3.33	9.98	0.9994	2.85	9.98
C2	8.06~128.93	0.9972	1.35	2.02	0.9969	1.61	3.22	0.9962	2.69	4.03

**Table 3:** Recovery, intra-day repeatability and inter-day reproducibility of the developed method in three matrixes *.

PSP Toxins	Spike Conc. (μg kg^−1^)	Scallop				Mussel				Clam			
		Intra-Day (%)		Inter-Day (%)		Intra-Day (%)		Inter-Day (%)		Intra-Day (%)		Inter-Day (%)	
		Recovery	RSD	Recovery	RSD	Recovery	RSD	Recovery	RSD	Recovery	RSD	Recovery	RSD	
STX	49.61	96.78	10.27	93.25	2.28	98.34	6.43	96.19	0.63	95.56	5.65	94.58	1.53
	26.46	89.74	9.76	100.30	5.37	92.43	5.06	96.73	4.22	97.80	6.18	95.86	0.35
	9.92	98.77	5.39	95.13	2.88	92.21	2.80	94.62	4.35	85.89	7.76	89.01	5.47
NEO	51.71	88.70	9.23	92.25	7.21	84.04	6.70	92.29	7.31	93.11	10.95	101.12	14.77
	27.58	90.54	14.63	91.84	14.39	88.92	12.01	83.62	6.22	90.93	3.63	94.33	0.56
	10.34	102.96	9.23	97.41	3.98	102.13	13.48	92.20	3.07	91.41	3.18	99.29	4.64
GTX1	62.11	84.69	14.34	89.96	5.09	99.56	9.86	96.50	3.45	96.01	12.53	97.80	9.45
	33.12	85.59	12.01	90.98	12.63	91.02	12.50	93.48	9.19	80.22	1.17	90.12	1.38
	12.42	89.18	18.17	96.42	4.05	88.28	16.19	97.87	2.42	89.32	19.10	87.66	0.62
GTX2	112.86	94.10	4.89	87.04	3.35	91.38	8.93	94.50	7.02	100.76	4.52	97.68	11.77
	60.19	95.32	16.22	89.71	3.22	99.78	7.20	96.46	1.61	116.53	7.32	85.87	13.25
	22.57	101.78	2.75	88.47	12.44	100.08	5.41	92.88	18.37	82.22	14.26	94.50	5.27
GTX3	47.89	96.18	2.24	90.08	3.88	82.80	5.62	94.74	2.74	99.39	1.50	90.38	1.12
	25.54	90.88	6.57	98.80	6.90	96.96	12.29	98.02	0.59	100.06	11.07	94.22	2.67
	9.58	92.36	8.62	93.96	17.47	86.36	5.05	97.04	0.59	93.60	13.00	99.01	6.32
GTX4	20.25	81.52	14.57	95.34	4.07	93.45	17.85	86.76	4.34	91.79	8.25	96.80	7.09
	10.80	83.66	12.66	89.00	7.72	82.63	12.51	95.46	8.85	95.84	6.83	95.16	1.74
	4.05	96.90	11.83	93.84	7.22	91.57	5.27	96.93	5.63	90.19	5.27	86.40	6.68
C1	134.78	89.06	13.85	93.98	2.90	90.19	11.26	94.37	2.42	107.86	9.87	98.09	1.13
	71.88	99.69	5.31	90.92	16.92	98.49	10.07	101.09	3.48	94.68	16.59	98.42	9.33
	26.96	94.82	10.19	97.59	11.21	96.91	11.23	88.36	7.38	86.42	3.20	94.23	1.69
C2	40.29	91.57	12.77	88.07	1.08	82.27	8.57	99.64	1.99	102.35	7.33	92.53	7.78
	21.49	96.45	18.70	86.50	9.04	104.50	15.53	100.67	1.71	96.18	8.07	96.78	8.27
	8.06	97.10	2.96	104.68	11.59	90.34	6.96	94.41	10.59	93.40	17.49	94.80	4.52

* Three levels of concentration, five days of consecutive analyses and six replications each day. RSD = relative standard deviation.

**Table 4:** Occurrence of PSP toxins in 30 collected shellfish samples (μg·kg^−1^).

Shellfish	Sample No.	Origin	STX	NEO	GTX1	GTX2	GTX3	GTX4	C1	C2
Scallop	1	Dalian	- *	-	-	-	-	-	-	-
	2	Qinhuangdao	-	-	-	-	-	-	-	-
	3	Tianjin	-	-	-	-	-	-	-	-
	4	Yantai	-	-	-	-	16.35	-	-	-
	5	Dalian	-	-	-	-	-	-	-	-
	6	Quanzhou	-	-	-	-	-	-	-	-
	7	Ningbo	-	-	-	7.85	-	-	-	-
	8	Qingdao	-	-	-	-	-	12.31	-	-
	9	Rizhao	-	-	-	-	-	-	-	-
	10	Xiamen	-	-	-	-	-	-	16.32	-
Clam	11	Lianyungang	-	-	-	-	6.24	-	-	-
	12	Dalian	-	-	-	-	-	-	-	-
	13	Qinhuangdao	-	-	-	-	-	-	-	-
	14	Tianjin	-	-	-	-	-	-	-	-
	15	Yantai	-	-	-	-	-	-	-	-
	16	Dalian	-	-	-	-	-	-	-	-
	17	Quanzhou	-	-	-	-	-	-	-	-
	18	Ningbo	-	-	-	-	15.68	-	-	-
	19	Qingdao	-	-	-	-	-	-	-	-
	20	Rizhao	-	-	-	-	-	-	-	-
Mussel	21	Ningbo	-	-	-	-	-	-	-	-
	22	Qingdao	-	-	-	-	11.44	20.82	-	-
	23	Rizhao	-	-	-	-	6.34	-	-	-
	24	Quanzhou	-	-	-	-	-	-	-	-
	25	Qinhuangdao	-	-	-	-	-	-	-	-
	26	Tianjin	-	-	-	-	9.87	-	-	-
	27	Yantai	-	-	-	-	-	-	-	-
	28	Taizhou	-	-	-	-	-	18.91	-	-
	29	Quanzhou	-	-	9.67	-	-	-	-	-
	30	Dalian	-	-	-	-	-	-	-	-

* Not detected.
